# Regulation of GLUT4 and Insulin-Dependent Glucose Flux

**DOI:** 10.5402/2012/856987

**Published:** 2012-10-17

**Authors:** Ann Louise Olson

**Affiliations:** Department of Biochemistry and Molecular Biology, University of Oklahoma Health Sciences Center, P.O. Box 26901, BMSB 964, Oklahoma City, OK 73190, USA

## Abstract

GLUT4 has long been known to be an insulin responsive glucose transporter. Regulation of GLUT4 has been a major focus of research on the cause and prevention of type 2 diabetes. Understanding how insulin signaling alters the intracellular trafficking of GLUT4 as well as understanding the fate of glucose transported into the cell by GLUT4 will be critically important for seeking solutions to the current rise in diabetes and metabolic disease.

## 1. Insulin-Dependent Glucose Flux

 The major physiologic action of insulin is to instruct tissues in the assimilation of dietary macronutrients and activate anabolic pathways that support growth and repair of tissues during the fed state. The actions of insulin on specific tissues vary depending both on the role that tissue plays in the assimilation of nutrients during the fed state, as well as the role that the tissue plays in the distribution of nutrients in the fasted state. The fasted state is defined as the period of time when the intestinal tract is no longer a significant source of nutrition. In the fed state, nutrients are being digested, absorbed and delivered to the body from the intestinal tract. In the fasted state, fatty acids and glycerol are released from adipose tissue, amino acids from skeletal muscle, and glucose from the liver. Therefore, liver, adipose tissue, and skeletal muscle, each play a very important role in exogenous nutrient assimilation after a meal, and the redistribution of endogenous nutrients during fasting.

 While insulin regulates the assimilation and distribution of all nutrients, insulin action is generally quantified through changes in glucose homeostasis. Insulin action also regulates amino acid uptake, protein synthesis, fatty acid uptake, fatty acid synthesis, and cholesterol synthesis through direct actions on the pathways that regulate these processes. The assessment of insulin sensitivity is only predicted through comparisons of blood glucose and blood insulin levels [1]; more, specifically, insulin resistance is inferred by observation of elevated plasma glucose levels following an overnight fast. The prevailing insulin levels in the fasted state reflect the Beta-cell response to hepatic glucose production. Plasma insulin levels in the fasted state will, in turn, feedback to modulate hepatic glucose production [2]. Thus, understanding the disposal of dietary glucose after a meal, and the regulation of hepatic glucose production during fasting are of considerable interest in the treatment and prevention of insulin resistance and type 2 diabetes.

 After consuming a full-meal, dietary glucose is taken up into peripheral organs through facilitated transport processes mediated by tissue-specific facilitative glucose transporters. For example, the rat liver takes up about 7% of glucose after a full meal, and this is largely due to transport by the low affinity, GLUT2 glucose transporter. GLUT2 is capable of transporting glucose over the large range of glucose concentrations found in the portal circulation following a meal [3–5]. Adipose tissue takes up another 7%, while skeletal muscle takes up 69% [3]. Heart, which accounts for only 1.2% of dietary glucose disposal, also utilizes an insulin-dependent glucose transport process [3]. 

 In contrast to GLUT2-dependent glucose transport in the liver, adipose, and skeletal muscle have an insulin-dependent glucose transport system that is responsible for postprandial glucose disposal in these tissues. It was known that insulin treatment of isolated rat adipocytes increased the Vmax for glucose transport by around 10-fold while the Km was unchanged [6], suggesting that enhanced glucose transport was either due to release of an inhibitor from the transporter itself or an increase in the number of transporters on the cell surface. The latter mechanism for insulin-dependent glucose transport was first supported by studies using isolated adipocytes [7, 8]. These two laboratories independently determined that insulin signaled the release (or translocation) of glucose transporters from an intracellular membrane compartment to the cell surface without changing the affinity of the transporters for binding glucose [7, 8]. This mechanism for insulin-dependent glucose uptake still stands.

 Eight years after the translocation hypothesis for insulin-dependent glucose uptake was proposed, a putative glucose transport protein from rat adipocytes was identified and shown to translocate from an intracellular pool to the cell surface in response to insulin [9]. Within a year, the cDNA encoding this protein was independently cloned by three laboratories [10–12], and subsequently referred to as GLUT4, the fourth member of the superfamily of facilitative glucose transporters. The identification and cloning of GLUT4 was the pivotal step in confirming the translocation hypothesis set forth nearly a decade earlier.

 Recently, another insulin-regulatable glucose transporter, GLUT12, has been identified and shown to enhance insulin-sensitivity in an overexpression model [13, 14]. Like GLUT4, GLUT12 also translocates to the cell surface of myocytes in response to insulin [15]. It is unclear if GLUT4 and GLUT12 have overlapping functions; however, it is unlikely, given the fact that GLUT4 knockout mice have a profound metabolic phenotype that is not compensated for by the presence of endogenous GLUT12 [16]. It remains to be determined what contribution GLUT12 makes to insulin-mediated glucose clearance.

## 2. GLUT4 Vesicle Traffic 

 Since the identification and cloning of GLUT4, a substantial effort has been devoted to understanding GLUT4 translocation and defining the nature of the intracellular pool of GLUT4 vesicles. The pool of GLUT4 vesicles that responds to insulin is biochemically distinct from GLUT4 vesicle populations that redistribute to the plasma membrane under other stimuli such as GTP*γ*S [17] or exercise [18, 19]. GLUT4 vesicles were first identified as part of the endosomal compartment by immunolocalization and colocalization with endosomal markers such as the transferrin receptor [20–23].

 In adipocytes, the GLUT4 storage (or retention) compartment is formed early in differentiation, even before GLUT4 is synthesized to significant levels [24]. Newly synthesized GLUT4 can be targeted directly to the plasma membrane, from which it gains entry into the early endosome [25]. Additionally, newly synthesized GLUT4 can fill the GLUT4 storage compartment from the transgolgi network (TGN) without first reaching the plasma membrane [26]. The insulin responsive pool of GLUT4 vesicles forms as a specialized postendocytic compartment that is separate from the recycling endosome pool [23, 27–29]. After insulin stimulation, internalized GLUT4 is sorted from the endosome to a specialized subcompartment of the TGN to the GLUT4 storage vesicles (GSVs) for retention [30]. GLUT4 Storage Vesicles (GSVs) are small, approximately 50 nm in diameter, and contain few other proteins than GLUT4 [31]. Insulin-responsive aminopeptidase (IRAP), GLUT4, and the v-SNARE, Vamp2, are the major components of the specialized insulin responsive pool of GSVs [32]. The lack of transferrin receptor (TfR) and other recycling endocytic components has established the specific nature of GSVs as a specialized postendocytic compartment that has a higher insulin responsiveness than regular recycling endosomes and can also contain GLUT4 [32].

 Kinetic studies demonstrate that the rate of GLUT4 exocytosis is accelerated by insulin treatment [29, 33–36]. Increased exocytosis may result from increased fission of GLUT4 vesicles from an endosomal precursor, stimulation of the rate of movement of vesicles to the plasma membrane, increased activation of docking and/or fusion machinery at the plasma membrane, or a combination of two or more of these processes. While many steps in the GLUT4 itinerary have been hypothesized to be insulin dependent, none of the processes or their molecular mechanisms have been firmly identified as targets of insulin signaling. Current thinking in the field has suggested that insulin may regulate either docking of GLUT4 vesicles with the plasma membrane, the fusion machinery such as SNAREs, or trafficking of GLUT4 to the cell surface/plasma membrane [26, 31, 37, 38].

 The translocation hypothesis suggested that insulin stimulation would increase the trafficking of GSVs to the cell surface. Studies attempting to link the insulin-signaling cascade to specific trafficking steps have been focused on the role of cytoskeleton or the release from a molecular tether [39, 40]. However, the observations that GSVs are highly mobile in the absence of insulin and that docking/fusion may be the important regulated steps, make it unlikely that insulin-dependent changes in vesicle movement are limiting for insulin-dependent GLUT4 redistribution to the cell surface [41–44]. This important shift in understanding, that the docking and fusion steps may be the key inputs of insulin signaling may lead to understanding the limiting interaction between insulin-signaling pathways and GLUT4 translocation.

## 3. Insulin-Signaling to GLUT4

 Insulin-signaling is initiated by the peptide hormone insulin binding to the *α*2-*β*2-heterotetrameric insulin receptor located on the cell surface (for review see [45]). The activated insulin receptor with a full complement of phosphorylated tyrosines can act as a docking site for insulin receptor substrate proteins (IRS 1/2) [46]. These adapter proteins recognize phosphorylated tyrosine residues on the receptor leading to a stable association between the receptor and the IRS proteins. Stable association leads to tyrosine phosphorylation of the IRS proteins by the insulin receptor *β*-subunit [47]. The importance of the IRS2 isoform in insulin mediated GLUT4 redistribution has been demonstrated by the specific knockout in mice, which develop severe insulin resistance [48]. IRS1 knockout mice were growth retarded, but an increase in insulin secretion prevented development of diabetes [48]. In addition, biochemical evidence from isolated brown adipocytes has supported the essential role for IRS2 in insulin stimulated GLUT4 redistribution [49]. While the IRS proteins contain no intrinsic enzyme activity, they are essential scaffolding molecules that bridge insulin receptor activity to downstream targets [50].

 The tyrosine phosphorylation sites on IRS 1/2 can act as docking site for recruitment of phosphatidylinositol 3-kinase (PI3-K) to the cell surface [51]. The importance of PI3-K activation in insulin-stimulated GLUT4 redistribution has been firmly established [52]. Relocation of PI3-K to the cell surface allows the enzyme to act on lipid substrates, in particular phosphatidylinositol 4,5-bisphosphate (PIP2), in the plasma membrane causing the generation of phosphatidylinositol 3,4,5-trisphosphate (PIP3). Generation of PIP3 patches in the plasma membrane can act as docking sites for plekstrin homolog (PH) domain containing proteins including protein kinase B (PKB), also known as Akt [45]. 

 Akt activation is crucial for insulin signaling to GLUT4 redistribution [53]. Akt is present in three isoforms with Akt1 contributing largely to growth, and Akt2 contributing to insulin-mediated GLUT4 redistribution [54]. Akt3 is not expressed in insulin-responsive tissues [55]. A constitutively active Akt (myr-Akt) redistributes GLUT4 to the plasma membrane in the absence of insulin and the Akt2 knockout mouse develops insulin resistance and type II diabetes [56, 57]. In order to become activated, Akt is recruited to the plasma membrane by its N-terminal PH domain. 

 Akt activation upon membrane recruitment is dependent on two phosphorylation events, one on threonine 308 and one a serine 473. Both are necessary for maximal Akt enzyme activity [58]. Threonine 308 phosphorylation is mediated by phosphoinositide-dependent protein kinase-1 (PDK1), which is also recruited to the plasma membrane by interaction with PIP3 [59]. The identification of the serine 473 kinase, also known as phosphoinositide-dependent protein kinase-2 (PDK2), has been an extremely controversial area with many proteins identified as harboring this activity [60]. Evidence has emerged that the PDK2 activity is fulfilled by the mTORC2 complex, which mediates serine 473 phosphorylation *in vivo* [61]. This result has been confirmed in 3T3-L1 adipocytes, a frequently used model for insulin-mediated GLUT4 redistribution [62]. A possible mechanism for activation of mTORC2 by insulin-signaling has not yet been identified, however, a recent report demonstrates that activated PI3-K recruits the mTORC2 complex to ribosomes, activating the complex [63]. In 3T3-L1 adipocytes, PDK2 activity has been isolated from a cellular compartment containing cytosketal components associated with the plasma membrane, but it is not known if ribosomes are also found in that compartment [64]. Interestingly, PDK2-dependent serine 473 phosphorylation precedes PDK1-dependent threonine 308 phosphorylation, and is important for maximal activation by PDK1 [61, 65]. The importance of PDK2 activity was clearly demonstrated in mice with a fat cell-specific ablation of *Richtor*, an important member the mTORC2 complex. These mice were shown to have defects in both lipid and glucose metabolism, presumably related to insulin-dependent GLUT4 translocation [66]. 

 Recently, AS160 (Akt substrate of 160 kDa) has been identified as a downstream target of insulin-stimulated Akt activity [67]. Sequence analysis predicted that AS160 was a Rab GTPase activating protein (GAP) domain. Rabs are known to be importantly involved in vesicle movement and fusion [68]. AS160 is phosphorylated by Akt on multiple residues and these phosphorylations have been hypothesized to lead to deactivation of the GAP function of the protein. Indeed, AS160 has been shown to be required for insulin-mediated GLUT4 redistribution and when Akt phosphorylation sites on AS160 are mutated, insulin is no longer able to signal GLUT4 redistribution [69]. Mutations of the GAP domain of AS160 suggest a functional requirement for GAP activity driving GLUT4 redistribution to the cell surface [69]. Knockdown of AS160 using siRNA confirms an inhibitory role for this protein in insulin-mediated GLUT4 redistribution [70]. In adipocytes, Rab10 is thought to be the primary target of AS160, although the broad substrate specificity for AS160 means that others Rabs may also be regulated [71, 72]. Although multiple Rab isoforms are required for GLUT4 intracellular traffic, Rab10 is the Rab-isoform that is required for insulin-mediated GLUT4 exocytosis [72, 73].

## 4. Regulation of Glucose Transport *In Vivo*

 Translocation of GLUT4 to the cell surface is clearly a major part of the underlying molecular mechanism responsible for the insulin-mediated increased Vmax of glucose transport [6]. Is GLUT4 translocation rate-limiting for glucose transport? The answer depends on the physiologic condition and the tissue being examined. For example, skeletal muscle glucose uptake depends on the coordination of three steps: (1) increased delivery of glucose to the muscle fiber by increased blood flow, (2) increased glucose transport across the plasma membrane (GLUT4 and other facilitative transporters), and (3) phosphorylation of glucose by hexokinase [74]. When grown in culture, the delivery of glucose to the cell surface of isolated myocytes is not limiting. The energy demands in culture are lower, so the overall rate of glucose transport is reduced, in turn, reducing stress on phosphorylation. Therefore, in the isolated myocyte, glucose transport is clearly rate limiting, although this may not be the case *in vivo*. In animals, blood glucose levels may vary depending on the physiologic environment. Glucose phosphorylation may be rate-limiting, especially under conditions where glucose flux is very high. For example, strenuous exercise increases blood flow, and glucose transport to a level that requires an increase in hexokinase expression [75]. Even so, transgenic overexpression of hexokinase II increased glucose uptake in exercised mice when GLUT4 levels were normal, but not in mice with ablation of a single allele of the GLUT4 gene [76]. These studies and others drive home the importance of the use of *in vitro* and *in vivo* models to understand the role of GLUT4 and glucose transport kinetics in normal and pathophysiologic states.

 Transgenic manipulation of GLUT4 levels has revealed that GLUT4 is, indeed, rate-limiting for insulin-dependent glucose uptake [77–79]. Moreover, the apparent changes in GLUT4-dependent glucose flux can alter the function of individual metabolic organs. The changes, such as those in the heart, are tissue autonomous and partially a result of altered whole-body metabolism. GLUT4 ablation in heart, as a result of either whole body ablation or targeted, cardiac-specific ablation, resulted in cardiac hypertrophy associated with increased size of myocytes, although the severity of the conditions differed between the two models [80, 81]. For example, the cardiac-specific GLUT4 knockout resulted in less severe cardiac hypertrophy compared to whole body-knockout. The excessive hypertrophy of the GLUT4 null mice is likely due to the fed hyperinsulinemia found in these animals. Complementation of skeletal muscle GLUT4 in the null mice reduced hyperinsulinemia and cardiac hypertrophy [79]. In contrast to cardiac hypertrophy, both models showed an upregulation of GLUT1, an apparent stress response to the decrease in insulin-dependent glucose uptake. It is likely that the cardiac adaptations are a result of a change in substrate availability to the cardiomyocyte. With the loss of insulin-dependent glucose flux, the predominant cardiomyocyte energy substrate would be fatty acid. The metabolic fate of glucose in the cardiomyocyte may contribute to the cytosolic redox state, as the ratio of reduced to oxidized glutathione is lower in the cardiac specific GLUT4 knockout mice [82]. The authors of this report treated mice with an antioxidant, Tempol, which reduced cardiac hypertrophy in the knockout mice. In this study, antioxidant treatment did not normalize the reduced glutathione levels, suggesting that the Tempol-mediated change in cardiac hypertrophy may have been an off-target effect. 

 Predictably, GLUT4 regulation plays a role in obesity-induced insulin resistance, which is characterized by increases in blood glucose and plasma insulin. During expansion of adipose, another important site of dietary glucose disposal, GLUT4 mRNA, and protein expression are reduced in adipose tissue [83, 84]. This may represent an important adaptive response for protecting the brain against hypoglycemia. As the adipose mass expands, if GLUT4 levels increased proportionally, glucose clearance by adipose tissue would also increase, diverting substrate from the nervous system. GLUT4 reduction leads to insulin-resistant glucose transport in this tissue [83–87]. The loss of GLUT4 in adipose tissue changes the function of this tissue in such a way that it impacts whole-body glucose homeostasis. For example, transgenic mice carrying a conditional, fat-specific knockout of GLUT4 had significantly impaired insulin action in muscle and liver [88]. These observations demonstrated a central role for GLUT4 levels in adipose tissue participating in whole-body glucose homeostasis and metabolic control.

 Skeletal muscle is the major site of dietary glucose disposal in the body [89]. The fate of GLUT4-mediated glucose flux in skeletal muscle appears to be metabolized through glycolysis and glycogen synthesis, at least in mice engineered to moderately overexpression the human GLUT4 gene [90]. Transgenic ablation of GLUT4 in muscle also results in insulin resistance and impaired glucose tolerance [91]. In insulin-resistant states, glucose transport into skeletal muscle was impaired [92, 93]. Reduced glucose uptake does not result from depletion of GLUT4 levels, but rather from inhibition of GLUT4 redistribution to the cell surface [94, 95]. The fact that the GLUT4 pool size in skeletal muscle is not reduced during insulin resistance and diabetes, as is observed in adipose tissue, allows for skeletal muscle to undergo exercise-induced GLUT4 translocation under conditions where there is a rapid demand for ATP in the working muscle [96]. The importance of GLUT4-mediated glucose flux in working muscle was clearly demonstrated in GLUT4-null mice stressed with 45 minutes of hypoxia. Under hypoxia, the GLUT4-null mice exhibited a significant decrease in ATP synthesis [97]. GLUT4-null mice were also slower to replenish glycogen stores following an exhaustive bout of swimming [98]. Interestingly, there was no compensatory glucose uptake mechanism invoked in the GLUT4 null mice subjected to hypoxia, while the bout of swimming did increase muscle-specific glucose uptake in the null mice [97, 98]. It would appear that hypoxia and exercise alter metabolism through different pathways, both of which rely on GLUT4, at least in part, for glucose flux to meet metabolic demands.

 Even though insulin resistance and diabetes do not reduce the pool of GLUT4 molecules in skeletal muscle, muscle-specific transgenic expression of GLUT4 significantly improved insulin action in diabetic mice [99]. Transgenic overexpression of GLUT4, either under the control of the native promoter or a tissue-specific, heterologous promoter, led to enhanced insulin sensitivity, enhanced glucose clearance, and provides some protection against insulin resistance. Altogether, these observations of GLUT4 have led most authors to conclude that upregulating glucose transporters may be an effective approach to the treatment of insulin resistance and human type 2 diabetes [83, 99–103]. 

 Moving forward with the notion that GLUT4 can be targeted as a therapeutic intervention for insulin resistance is limited by the fact that we do not fully understand the physiologic impact of GLUT4 on metabolic regulation. For example, transgenic expression of GLUT4 in adipose tissue, under the control of the AP2 promoter, had increased serum triacylglycerol and free fatty acids [104]. Such lipid abnormalities are consistent with enhanced mobilization of lipids and higher than normal rates of betaoxidation in liver. Along with increases in insulin and glucose, increased free fatty acids are manifested in obese conditions. However, the basis for abnormalities in lipid metabolism is not clearly understood, and the source of the serum lipids has not been identified within these GLUT4 overexpressing models. To confuse this issue further, many of the transgenic overexpression studies have been performed with tissue-specific, heterologous promoters which do not undergo the same regulatory responses to hormonal and metabolic signals as does the GLUT4 promoter [78, 105]. For example, overexpression of GLUT4 in adipose tissue driven by the AP2 promoter leads to an abnormally high level of GLUT4 overexpression that is not susceptible to that downregulation of GLUT4 expression that would be observed in models of insulin resistance. Adipose tissue in these animals have upregulated expression of the carbohydrate response element binding protein (ChREBP) beta-isoform which converts excess dietary carbohydrate to fat in these mice [106]. The caveat to this study is that ChREBP-beta would only be induced as a result of massive overexpression of GLUT4 and, without that, the flux of glucose, even in diet-induced obesity would never be high because endogenous GLUT4 expression would be decreased. 

 To resolve the gene expression problem, an hGLUT4 transgenic model was established in 1993, and has been well characterized [77, 90, 107]. The plasmid used to generate this line of mice consisted of the entire coding region of the hGLUT4 under the control of its natural promoter. The level of overexpression is moderate, within a range that might be anticipated from a chronic exercise routine [108–110]. Moreover, this transgenic construct is subject to the expected hormonal and metabolic regulation of expression [107], making this model a highly useful representation for studying how GLUT4 overexpression modifies adaptation to metabolic stresses, such as diet-induced obesity and insulin resistance. 

 While the hGLUT4 transgenic (hGLUT4 TG) mice are highly insulin sensitive [90], they develop metabolic characteristics that are highly linked to type 2 diabetes in humans, including hepatic steatosis and obesity [111]. For example, transgenic expression of human GLUT4 (hGLUT4) under the control of its own promoter enhances insulin sensitivity, but at the same time significantly increases serum triacylglycerol levels, free fatty acids, and ketones [90]. These animals have not been well studied under conditions that result in insulin resistance. Therefore, we do not understand how lipid metabolism would be altered in type 2 diabetes in these animals.

 To observe the effects of GLUT4 overexpression under an insulin resistant state, the hGLUT4 transgenic model was interbred with genetically diabetic *db/db* mice, which is a model of obesity, diabetes, and dyslipidemia wherein leptin receptor activity is deficient. Data from this investigation demonstrated that moderate overexpression of GLUT4 improved glucose tolerance; however, glucose tolerance declined with age even though muscle tissue retained enhanced insulin-dependent glucose uptake [100, 112]. The decline in glucose tolerance was attributed to eventual atrophy of pancreatic islet cells, demonstrating that preserving muscle glucose uptake alone will not fully prevent development of diabetes [100]. An important, yet completely unexplained observation was the unexpected effect of the hGLUT4 transgene on fed insulin levels in the *db/db* background. The hGLUT4 interbred on the lean *db/+* background maintained low plasma glucose levels and low plasma insulin levels in both the fed and fasted states [112]. In contrast, when the hGLUT4 transgene was expressed in the obese db/db background, the fed and fasting glucose and insulin levels were much higher than in the lean *db/+* background. When compared to non-transgenic *db/db* mice, the hGLUT4 *db/db* had significantly lower fed glucose, and significantly higher fed insulin levels [112]. This observation suggests that the hGLUT4 *db/db* mice, an extreme model of obesity, experience significant insulin resistance in spite of the normal glucose tolerance. It is important to extend these studies to a more physiologic model of diet-induced obesity.

## 5. Regulation of GLUT4 Synthesis 

 As with all genes subject to restricted patterns of regulation, expression of *GLUT4* mRNA is subject to tissue-specific regulation. GLUT4 is also subject to developmental regulation as full expression of GLUT4 is not achieved until the perinatal period [113]. *GLUT4* mRNA expression is largely restricted to brown and white adipose tissue, skeletal muscle, and heart. As described above, glucose uptake via GLUT4 in these tissues is used for very different purposes related to the tissue's function. 

 GLUT4 gene expression is also subject to up and downregulation depending on the physiologic state of the organism. Changes in *GLUT4* gene expression are observed in physiologic states of altered glucose homeostasis. In general, *GLUT4* mRNA expression is reduced in such states of severe insulin deficiency, such STZ-induced diabetes and nutritional deprivations such as starvation (for review see [114, 115]). These changes in steady-state *GLUT4* mRNA levels are tissue specific. For example, changes in *GLUT4* mRNA expression occur much more rapidly in adipose tissue than skeletal muscle [116]. Chronic fasting markedly reduces *GLUT4* mRNA levels in adipose tissue, but has little or no effect on *GLUT4* mRNA in skeletal muscle [117]. These tissue-specific physiologic adaptations are consistent with the tissue-specific fates of GLUT4-dependent glucose uptake in these tissues. Specifically, muscle must retain its pool of GLUT4 so that it can call on GLUT4 to respond to exercise and muscle contraction. 

 Changes in steady-state levels of *GLUT4* mRNA could potentially be the result of changes in either the rate of synthesis of *GLUT4* mRNA (gene transcription) or stability of messenger RNA. Nuclear run-on assays measuring the rate of *GLUT4* mRNA transcription demonstrate that transcription is decreased in both adipose tissue and skeletal muscle in STZ-induced diabetic animals [118, 119], while the rate of the *GLUT4* gene transcription in skeletal muscle of fasted animals is increased [119]. Thus, changes in *GLUT4* mRNA steady-state levels reflect changes in the rate of mRNA synthesis. 

 In contrast to the downregulation of *GLUT4* which is observed in insulin deficiency and fasting, *GLUT4* expression is increased at the transcriptional level by endurance exercise [119]. The increase in *GLUT4* gene expression in response to exercise is rapid, occurring after only one session of aerobic exercise [120]. This increase is on the order of 1.5- to 2-fold, and it is enough to modify carbohydrate metabolism [120]. After cessation of endurance training, GLUT4 expression reverts to normal levels in two to four days [121, 122]. Activation of the AMP-activated protein kinase (AMPK) is known to occur during muscle contraction in response to increased AMP and decreased ATP and phosphocreatine. For this reason, activation of AMPK was thought to mediate the exercise-induced changes in GLUT4 expression; however, expression of dominant-negative AMPK does not inhibit exercise-induced increase in GLUT4 transcriptional activity [123]. Thus, the signaling cascades responsible for exercise-induced gene expression remain unknown.

 Regulation of GLUT4 gene expression within a very narrow range (2- to 3-fold) has a significant effect on whole-body glucose homeostasis, suggesting that a sort of rheostatic control of GLUT4 levels is an important adaptation to extreme physiologic states or chronic adaptation to pathologic states, such as obesity. Because this gene falls under complex metabolic control, the molecular basis for regulation of *GLUT4* gene expression in extreme physiologic states has been very difficult to determine. For example, the insulin-deficient state is complicated by the elevation of counter-regulatory hormones. Consistent with elevated levels of counter-regulatory hormones, an increase in intracellular cAMP has been shown to decrease GLUT4 gene expression [118, 124] In addition, insulin deficiency is tightly coupled to plasma glucose levels, intracellular glucose utilization, and intracellular lipid accumulation. Each of these factors may play a role in signaling the nucleus to modify levels of GLUT4 gene expression. Until we have unraveled the molecular mechanism that regulates GLUT4 promoter activity, we cannot understand how metabolic signals to the nucleus are transmitted.

## 6. Identification of Promoter Regulatory ****Elements

 To understand how the metabolic environment influences *GLUT4* gene transcription, it first necessary to identify the molecular elements (*cis*-DNA sequences and *trans*-acting factors) that regulate the gene. Traditionally, promoter studies are carried out in model cell culture systems, owing to their ease of transfection and experimental manipulation. The general strategy of these studies is to clone specific segments of a given promoter upstream of an easily measured reporter gene, followed by transfection into a suitable cell line. Levels of reporter mRNA or protein are then used as indirect measurements of the transcriptional activity of the promoter fragment under study. This approach did not work for analyzing the GLUT4 promoter. Shortly after the cloning and identification of the human and rodent GLUT4 genes, multiple labs began to analyze the GLUT4 gene promoter. Tissue culture models were limited due to the fact that GLUT4 gene expression is developmentally regulated, and can only be studied in differentiated cultured myotubes and adipocytes [125–127]. Early work using differentiated C2C12 myoblasts identified an authentic Myocyte Enhancer Factor 2 (MEF2) binding domain to be required for differentiation-dependent gene expression [128, 129]. In cultured adipocytes, differentiation-dependent gene expression is dependent on the MEF2-binding domain, and a second DNA-binding site found a few hundred base pairs upstream of the MEF2 domain [130]. The second binding site, known as Domain 1, binds a variety of transcription factors [130, 131]. Differentiation-dependent GLUT4 gene expression is not solely regulated by the binding of transactivating factors to cognate DNA-binding sites. Instead, differentiation-dependent GLUT4 gene expression is regulated by the release of class II histone deactyleases (HDACs) that bind to the transactivating factors [132, 133]. The class II HDACs are abundant in the nucleus of undifferentiated preadipocytes [133]. As the cells undergo differentiation, the class II HDACs redistribute to the cytoplasm, allowing GLUT4 mRNA synthesis to proceed.

## 7. Analysis of the Human GLUT4 Promoter *In Vivo*

 The value of tissue culture systems in transcription promoter analyses is undisputed. However, additional information can be obtained by analyzing promoter activity in transgenic animals. In the case of *GLUT4*, we have observed a complex pattern of gene expression in various physiologic states, which are not possible to represent in tissue culture models. Also, transcription analysis in transgenic mice allows us to measure effects of specific promoter elements in a variety of tissues under the influence of various hormonal, nutritional, and metabolic factors.

**Figure 1:**
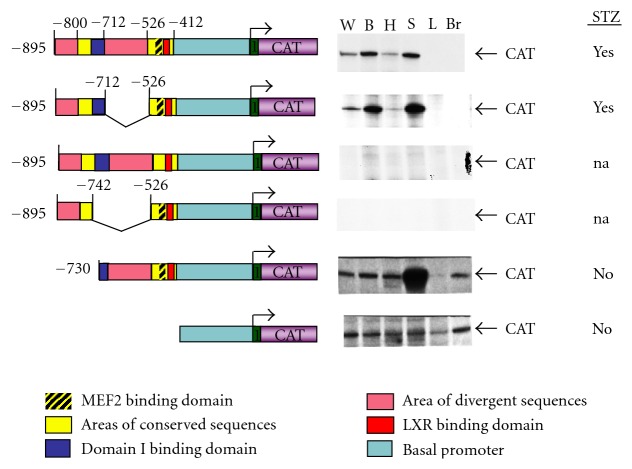
Summary of selected human GLUT4 promoter/CAT reporter constructs used to generate transgenic mice. The major regulatory domains (Domain 1, MEF2, and LXRE are shown. Expression of transgenic mRNA from white (W) and brown (B) adipose, skeletal muscle (S), heart (H), liver (L) and brain (Br). STZ indicates regulation under streptozotocin diabetes similar to endogenous. GLUT4.

 To date, we have analyzed 12 transgenic constructs for tissue-specific and hormonal-dependent *GLUT4* gene regulation (Figure 1). The first transgenic line was engineered to express a human *GLUT4* minigene consisting of the entire coding region of the gene plus 5.3 kb of 5′ flanking DNA [107]. Expression of this construct demonstrated that the human gene could function in a mouse background. This allowed us to generate a line of transgenic mice carrying a DNA construct in which the chloramphenicol acetyltransferase (CAT) reporter gene was expressed under the control 2.4 kb of human *GLUT4* DNA located immediately 5′ of the transcription start site [134].

A series of deletions in the 5′ end of the human *GLUT4* promoter allowed us to map important promoter regulatory elements to the first 895 bp upstream of the major transcription initiation site (Figure 1 and [135]). A comparison of sequences of the human, mouse, and rat GLUT4 gene promoter revealed highly conserved portions containing more than 90% sequence identity [136]. The most distal conserved area did not possess a binding site for any known transcription factors. We referred to this area as *Domain I*. The proximal conserved area contained a perfectly matched binding site for MEF2A/D transcription factors that we refer to this site as the *MEF2 domain*. The proximal area also contains a conserved *LXRE* 12 bp downstream of the MEF2 domain. The high-sequence conservation in these areas suggested that they were important functional sites for regulation of *GLUT4* gene transcription. Removal of the poorly conserved sequences (less than 85%) between these domains had no effect on transcriptional activity (Figure 1, constructs 1 and 2). A 5′ deletion of both domains (Figure 1, construct 7) left a basal promoter that expressed at a very low level in all tissues, including those that do not normally express *GLUT4* [137]. Also, a 5′ deletion through Domain 1 (Figure 1, construct 5) resulted in ubiquitous expression of the transgene, but with a slightly higher level of expression in skeletal muscle. Importantly, construct 5 did not support regulated expression of transgenic mRNA under conditions of STZ-deficiency, in skeletal muscle or any other tissues. Thus, by our criteria, the MEF2 domain per se does not constitute a fully functional promoter. The MEF2 domain is nevertheless required, since a mutation of this site completely ablated transgenic mRNA expression (Figure 1, construct 3). Thus, the MEF2 domain is necessary, but not sufficient for full function of the *GLUT4* promoter [135]. An internal deletion of only Domain I was also not expressed in mice (Figure 1, construct 4, and [138]). The difference in expression between constructs 4 and 5 suggest that sequences upstream of Domain 1 may repress transcription in some tissues. Thus, the MEF2 domain functions cooperatively with Domain 1 to support regulated transcription of the human *GLUT4* promoter. 

We, as well as others, have shown that these elements play both a positive role in tissue-specific expression and a negative role under certain physiologic states such as insulin deficiency [123, 137, 139]. To illustrate this point, transgenic animals described in Figure 1 were subjected to an STZ protocol to induce experimental diabetes and treated with insulin or left untreated, to determine how this affected transgene expression. CAT mRNA, driven by the truncated promoter fragments was compared to a fully functional human GLUT4 promoter and the endogenous mouse GLUT4 (Figure 1). CAT mRNA, which was driven by both 730 bp or 412 bp fragments of GLUT4 promoter DNA did not respond to either STZ treatment (Figure 1, constructs 5 and 6), or exercise treatment [123]. This shows that sequences somewhere upstream of −730 are required to create changes in gene expression in response to various physiologic state, just as these same sequences are responsible for restricting tissue-specific expression. These data indicate that *cis-acting DNA can play both a negative and positive role* in regulating GLUT4 promoter activity, depending on the other proteins present in the nucleus that are capable of interacting with the GLUT4 promoter. Notably, the class II HDACs play a role in both upregulation and downregulation of GLUT4 gene expression in mice in response to altered physiologic states. For example, the upregulation of skeletal muscle GLUT4 mRNA in response to exercise correlates with loss of HDAC 4 and HDAC 5 from muscle nuclei [140–142]. Conversely, the downregulation of GLUT4 mRNA in adipose tissue following diet-induced obesity is attributed to increased HDAC 4 and HDAC 5 association with the GLUT4 promoter in adipose nuclei [143]. The mechanism by which the class II HDACs associate with the GLUT4 promoter is still under investigation. The specific binding partner(s) have not been fully elucidated. It is known HDAC association with the GLUT4 promoter in adipose tissue is dependent on the presence of the LXRE which is immediately adjacent to the MEF2 site [143]. Further work is required to understand how the LXRE and its cognate binding partners regulate GLUT4 mRNA expression.

## 8. Conclusions

 The insulin-responsive facilitative glucose transporter, GLUT4, plays an extremely important physiologic role in the partitioning of glucose among peripheral tissues. In the normal cycle of fasting and refeeding, insulin is the key regulator of GLUT4 redistribution to the cell surface leading to increased glucose flux, yet the fate of glucose transported by GLUT4 is not completely understood. Presumably, GLUT4-mediated glucose flux leads to glucose entering both oxidative and nonoxidative metabolic pathways. Transgenic manipulation of GLUT4 has demonstrated that a variety of pathways are altered. A second important role for GLUT4 is to provide a mechanism of enhanced glucose uptake in working skeletal muscle. GLUT4 plays an important role in the adaptation of skeletal muscle to increased metabolic demand during periods of prolonged muscle contraction. It is this latter function that may explain the mechanism by which exercise promotes insulin sensitivity in healthy, insulin-resistant people. Partitioning of glucose to skeletal muscle may act to reduce blood glucose levels, prevent development of hyperinsulinemia, and prevent high levels of *de novo* fatty acid synthesis in obese and type 2 diabetic people, thus preventing the progression of disease.
